# Loss of TRIM44 promotes renal cell carcinoma progression by regulating K48-linked ubiquitination of vimentin

**DOI:** 10.1016/j.jbc.2025.110734

**Published:** 2025-09-16

**Authors:** Ke Shi, Baiyun Jia, Yuanyu Li, Xiaojuan Feng, Xusheng Sun, Qingjuan Liu, Wei Zhang, Yuexin Tian, Xinyan Miao, Yunhe Liu, Hang Zhao, Lihua Kang, Tongyu Zhao, Shiqi Zhang, Jinxi Liu, Shuxia Liu

**Affiliations:** 1 Department of Pathology, Hebei Key Laboratory of Nephrology, Center of Metabolic Diseases and Cancer Research, Hebei Medical University, Shijiazhuang, China; 2 Department of Oncology, The Fourth Hospital of Hebei Medical University, Shijiazhuang, China; 3 Department of Thoracic Surgery, The Fourth Hospital of Hebei Medical University, Shijiazhuang, China; 4 Department of Pathology, The Fourth Hospital of Hebei Medical University, Shijiazhuang, China; 5 Clinical College of Hebei Medical University, Shijiazhuang, China; 6 Department of Pathology, The First Hospital of Hebei Medical University, Shijiazhuang, China

**Keywords:** renal cell carcinoma, TRIM44, B-box domain, vimentin ubiquitination, progression

## Abstract

Tripartite motif-containing 44 (TRIM44), a member of the TRIM protein family, has emerged as a regulator in multiple cancer types, yet its functional role and molecular mechanisms in clear cell renal cell carcinoma (ccRCC) remain poorly characterized. Here, we identified TRIM44 as a tumor suppressor in ccRCC through integrated clinical and functional analyses. Clinically, TRIM44 expression was significantly downregulated in ccRCC tissues compared with adjacent normal tissues, and its reduced expression correlated with advanced tumor stage and poor patient prognosis. Functionally, gain-of-function and loss-of-function experiments demonstrated that TRIM44 potently inhibited ccRCC cell migration, invasion, and proliferation *in vitro* and *in vivo*. Mechanistically, TRIM44 directly interacts with vimentin. Importantly, we found that TRIM44 promotes K48-linked polyubiquitination of vimentin through its B-box domain, thereby targeting vimentin for proteasomal degradation. Collectively, our study establishes TRIM44 as a critical regulator of ccRCC progression through vimentin destabilization, highlighting its potential as both a prognostic biomarker and therapeutic target for ccRCC.

Kidney cancer is one of the 10 leading cancer types, according to Cancer Statistics 2023 (1). Clear cell renal cell carcinoma (ccRCC) is the most common subtype of renal cell carcinoma, accounting for approximately 75% of cases (2). Partial or radical nephrectomy is used to treat patients with early or localized ccRCC, with a 5-year survival rate of 92.6%. About one-third of patients, however, including those undergoing nephrectomy, are diagnosed at the metastatic stage (3), and despite several new approaches for metastatic ccRCC, the progression and metastasis of ccRCC remain unsolvable problems (4). The precise mechanism responsible is currently unclear. Thus, there is an urgent clinical need to discover a novel therapeutic target for metastatic ccRCC.

Ubiquitination, a critical post-translational modification, is mediated by sequential actions of E1 activating enzymes, E2 conjugating enzymes, and E3 ligases that attach ubiquitin chains to target proteins, thereby regulating their degradation or functional activation (5). As one of the largest E3 ligase families, TRIM proteins participate in diverse biological processes, including cancer progression, inflammation, autophagy, and intracellular signaling through their ubiquitin ligase activity (6, 7). Emerging evidence reveals the dual roles of TRIM family members in tumorigenesis, where TRIM3 functions as a tumor suppressor by sequestering p21 to inhibit cyclin D1-CDK4 accumulation (8), while TRIM17 promotes gastric cancer through BAX stabilization and apoptosis inhibition (9). Similarly, TRIM22 exerts tumor-suppressive effects in osteosarcoma by destabilizing nuclear factor erythroid 2-related factor 2 and activating ROS/AMPK/mTOR/autophagy signaling (10). TRIM44 demonstrates context-dependent oncogenic or tumor-suppressive functions across different cancers, including its role in lung adenocarcinoma (11), ovarian cancer (12), and gastric cancer (13) where it regulates lysyl oxidase-like 2 stability to remodel extracellular matrix and modulate tumor immunity (14). Despite these advances, the precise role and molecular mechanisms of TRIM44 in ccRCC progression remain to be elucidated.

Progression and metastasis are the ultimate and most deadly manifestations of cancer, and numerous studies have shown that most cancer patients die from metastatic disease rather than from the primary tumor (15). TRIMs is also been reported to play a vital role in tumor metastasis. For example, TRIM50 facilitated Snail1 degradation to reverse epithelial–mesenchymal transition (EMT) and inhibit pancreatic cancer progression (16), while Xiong *et al.* found that TRIM44 activated the Akt/mTOR signaling pathway and modulated the EMT process to promote the proliferation, migration, and invasion of esophageal cancer cells (17). Notably, tumor metastasis is associated with aberrant reactivation of EMT, which is regulated by a complex network, including transcription factors (Snail1/2, Twist1, and Zeb1/2), post-translational control, and noncoding RNA-mediated regulation, leading cells to switch between epithelial and mesenchymal states (18, 19, 20). However, it is unclear whetherTRIM44 can regulate the EMT processes to affect ccRCC progression, and clarifying its mechanism will provide a new direction for the treatment of ccRCC metastasis.

In this study, we investigated the relationship between TRIM44 expression and cancer progression in cell lines and in patients with ccRCC. We then elucidated the precise mechanism by which TRIM44 regulated ccRCC progression *in vivo* and *in vitro*.

## Results

### TRIM44 was downregulated in RCC

**Figure 1:**
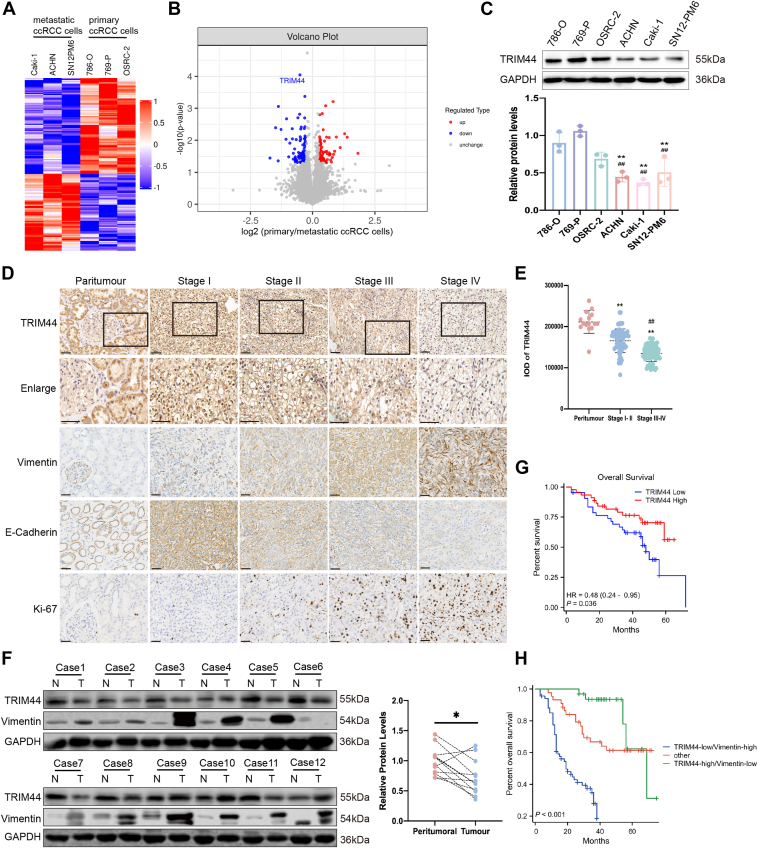
**TRIM44 was down-regulated in clear cell renal cell carcinoma (ccRCC) and associated with disease progression.***A*, Heatmap representation of comparative proteomic analysis between primary (786-O, 769-P, OSRC-2) and metastatic (ACHN, Caki-1, SN12-PM6) ccRCC cell lines. Fold change> 1.2 or <0.83, *p* < 0.05 was regarded as significant. *B*, Volcano plot demonstrating significant downregulation of TRIM44 in metastatic *versus* primary ccRCC cell lines. *C*, Expression intensity of endogenous TRIM44 in six ccRCC cell lines and statistical data graph (^∗^compared with 786-O, ^#^compared with 769-P). *D*, Representative IHC staining of TRIM44, vimentin, E-Cadherin and Ki-67 in ccRCC tissues. Scale bar: 50 μm. *E*, statistical data graph of TRIM44 expression in peritumour and different tumor stages (^∗^compared with Paritumour, ^#^compared with StageⅠ-Ⅱ). *F*, expression of TRIM44 and vimentin in 12 pairs of ccRCC samples. *G*, Kaplan–Meier survival analysis showing poorer overall survival in TRIM44-low *versus* TRIM44-high ccRCC patients (HMU-RCC cohort). *H*, combined survival analysis of TRIM44 and vimentin expression levels. ∗*p* < 0.05, ∗∗*p* < 0.01 and ^##^*p* < 0.01.

To determine the critical protein in metastatic ccRCC, we performed proteomics analysis using different ccRCC cell lines. There were 72 up-regulated and 90 down-regulated proteins in metastatic (ACHN, Caki-1, and SN12-PM6) compared with primary ccRCC cell lines (786-O, 769-P, and OSRC-2) (Fig. 1*A*). ACHN, Caki-1, and SN12-PM6 cell lines were obtained from pleural effusion, skin metastasis, and lung metastasis of ccRCC, respectively. TRIM44 was one of the most significantly down-regulated proteins in metastatic ccRCC cell lines compared with primary ccRCC cell lines (Fig. 1*B*). We further investigated the expression and significance of TRIM44 in ccRCC by detecting its expression in six cell lines and ccRCC tissues from 144 patients by Western blot and IHC, and we analyzed the correlations between TRIM44 expression and clinical pathological parameters. TRIM44 expression levels were lower in metastatic ccRCC than in primary ccRCC cell lines (Fig. 1*C*). In addition, IHC showed that TRIM44 was mainly localized in the nucleus and cytoplasm in ccRCC, and its expression was lower in ccRCC tissues compared with non-cancerous adjacent tissues (Fig. 1, *D*–*F*). Importantly, TRIM44 protein expression was downregulated in advanced-stage compared with early-stage tumors (Fig. 1*D*). Correlation analysis showed that TRIM44 expression was significantly negatively correlated with tumor size (*p* < 0.001), Fuhrman grade (*p* < 0.001), TNM stage (*p* < 0.001), lymph node metastasis (*p* = 0.019), distant metastasis (*p* = 0.001), and a poor prognosis of ccRCC (*p* < 0.001) (Table 1). The results of univariate and multivariate analyses are shown in Table 2. Univariate analysis identified TNM stage, tumor size, Fuhrman grade, lymph node metastasis, distant metastasis, and TRIM44 staining as factors associated with overall survival (OS), while multivariate analysis identified TRIM44 as an independent predictor for postoperative OS in patients with ccRCC. According to the clinical information for the 144 patients, the median OS of patients with high TRIM44 expression was higher than that of those with low TRIM44 expression (Fig. 1*G*). In conclusion, low expression of TRIM44 was identified as a prognostic marker in patients with ccRCC.

**Table 1:** Correlations between TRIM44 expression and clinicopathological factors

Characteristics	Number	TRIM44 expression		
		Low (n = 77)	High (n = 67)	*p* value
Age				
<60	76	39	37	0.583
≥60	68	38	30	
Gender				
Male	93	53	40	0.253
Female	51	24	27	
Tumour size (cm)				
≤7	42	10	32	**<0.001**^a^
>7	102	67	35	
Fuhrman grade				
I-II	87	35	52	**<0.001**^a^
III-IV	57	42	15	
TNM stage				
I-II	68	17	51	**<0.001**^a^
III-IV	76	60	16	
Lymph nodes metastasis				
Yes	32	28	4	**<0.001**^a^
No	112	49	63	
Distant metastasis				
Yes	30	24	6	**0.001**^a^
No	114	53	61	
Death				
Yes	65	51	14	**<0.001**^a^
No	79	26	53	
Recurrence				
Yes	46	29	17	0.115
No	98	48	50	

Bold values indicates statistical significance.

A chi-square test was used for comparing groups between low and high.

TRIM44 expression.

a *p* < 0.001.

**Table 2:** Univariate and multivariate analyses of factors associated with OS

Characteristics	Total (N)	Univariate analysis		Multivariate analysis	
		Hazard ratio (95% CI)	*p* value	Hazard ratio (95% CI)	*p* value
Age	144				
≥60	68				
<60	76	1.061 (0.648–1.738)	0.814		
Gender	144				
Male	93				
Female	51	0.825 (0.486–1.401)	0.477		
TNM stage	144				
I-II	68				
III-IV	76	9.363 (4.582–19.134)	**<0.001**^c^	3.753 (1.584–8.894)	**0.003**^b^
Tumour size	144				
>7	102				
≤7	42	0.344 (0.174–0.677)	**0.002**^b^	0.946 (0.431–2.072)	0.889
Fuhrman grade	144				
I-II	87				
III-IV	57	2.545 (1.549–4.179)	**<0.001**^c^	1.154 (0.686–1.942)	0.590
Lymph nodes metastasis	144				
Yes	32				
No	112	0.205 (0.123–0.340)	**<0.001**^c^	0.514 (0.295–0.896)	**0.019**^a^
Distant metastasis	144				
Yes	30				
No	114	0.228 (0.138–0.378)	**<0.001**^c^	0.529 (0.311–0.902)	**0.019**^a^
TRIM44	144				
Low	77				
High	67	0.190 (0.104–0.347)	**<0.001**^c^	0.488 (0.244–0.976)	**0.043**^a^

Bold values indicates statistical significance.

*p* value was calculated using Cox proportional hazards regression.

a *p* < 0.05.

b *p* < 0.01.

c *p* < 0.001.

The TNMplot (https://tnmplot.com/analysis/) and The Cancer Genome Atlas (TCGA) databases showed similar results (Fig. S1, *A*–C). Furthermore, another analysis of ccRCC survival status based on the TCGA database demonstrated that TRIM44 downregulation predicted decreased disease-free survival and OS benefits (Fig. S1, *D* and *E*). Overall, TRIM44 was a vital protein with an important role in ccRCC tumorigenesis and metastasis.

### TRIM44 restrained ccRCC progression *in vitro*

**Figure 2:**
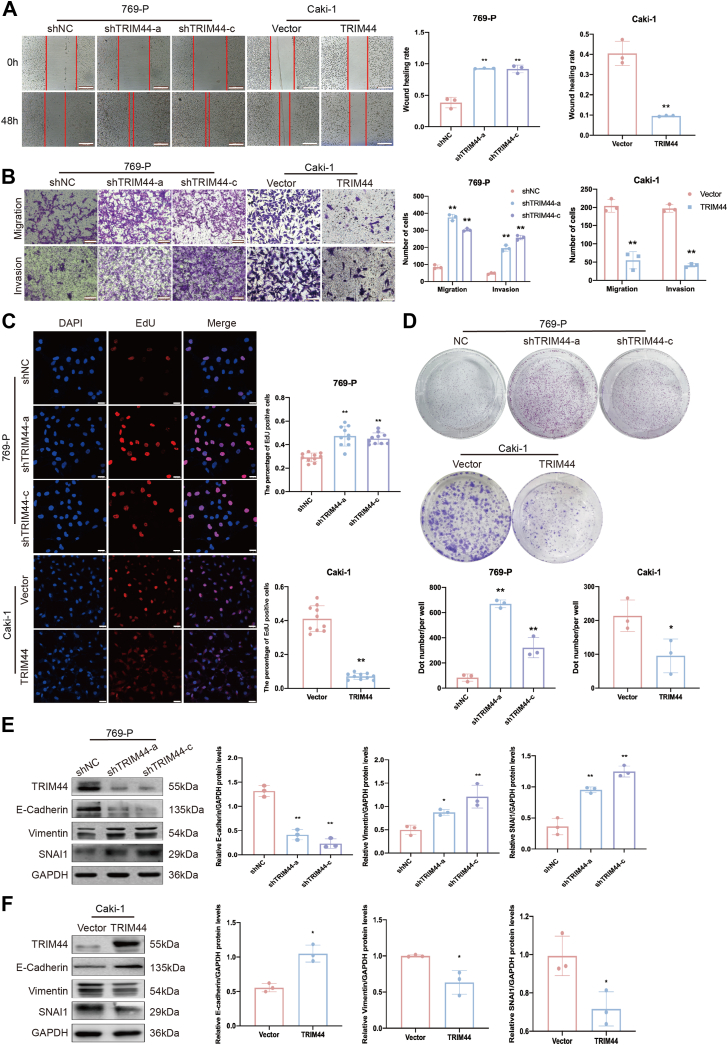
**TRIM44 restrained ccRCC progression *in vitro*.***A*–*D*, knockdown of TRIM44 enhanced cell migration, invasion, proliferation, and colony formation ability in 769-P cell lines; overexpression of TRIM44 reduced cell migration, invasion, proliferation, and colony formation ability in Caki-1 cell lines. *A*, cell wound scratch assay. Scale bar: 500 μm. *B*, Transwell migration and invasion assay. Scale bar: 100 μm. *C*, EdU proliferation staining, Scale bar: 25 μm. *D*, Colony formation assay. *E* and *F*, the expression of E-Cadherin, vimentin, snai1, and TRIM44 was detected by Western blot assay. ∗*p* < 0.05 and ∗∗*p* < 0.01.

We investigated the potential function of TRIM44 in ccRCC progression by transfecting 769-P and OSRC-2 cells with TRIM44 short hairpin RNAs, and transfecting Caki-1 and ACHN cells with a TRIM44-overexpression plasmid, and then we detected the transfection efficiencies by Western blot and real-time polymerase chain reaction (Fig. S2). In addition, the cells’ migration, invasion, and proliferation abilities were detected by scratch assay, Transwell assay, and EdU incorporation and clone assays, respectively. TRIM44 suppression enhanced the invasion, migration, and proliferation of 769-P cells and OSRC-2 cells, while upregulating TRIM44 attenuated those abilities in Caki-1 cells and ACHN cells (Figs. 2, *A*–*D*, S3, *A*–*D*).

EMT and stemness are vital processes in tumor invasion and migration (21). We further explored the possible mechanism of TRIM44 in the progression of ccRCC by detecting markers of EMT and stemness. Knockdown of TRIM44 resulted in low E-cadherin and high vimentin and Snail levels, while TRIM44 overexpression had the opposite effects (Fig. 2, *E* and *F*, Fig. S3, *E* and *F*); however, regulating TRIM44 expression did not affect the level of stemness marker (Fig. S4). Collectively, these results indicate that TRIM44 is critical for the EMT process in ccRCC cells.

### TRIM44 bound to vimentin in the cytoplasm in ccRCC cells

**Figure 3:**
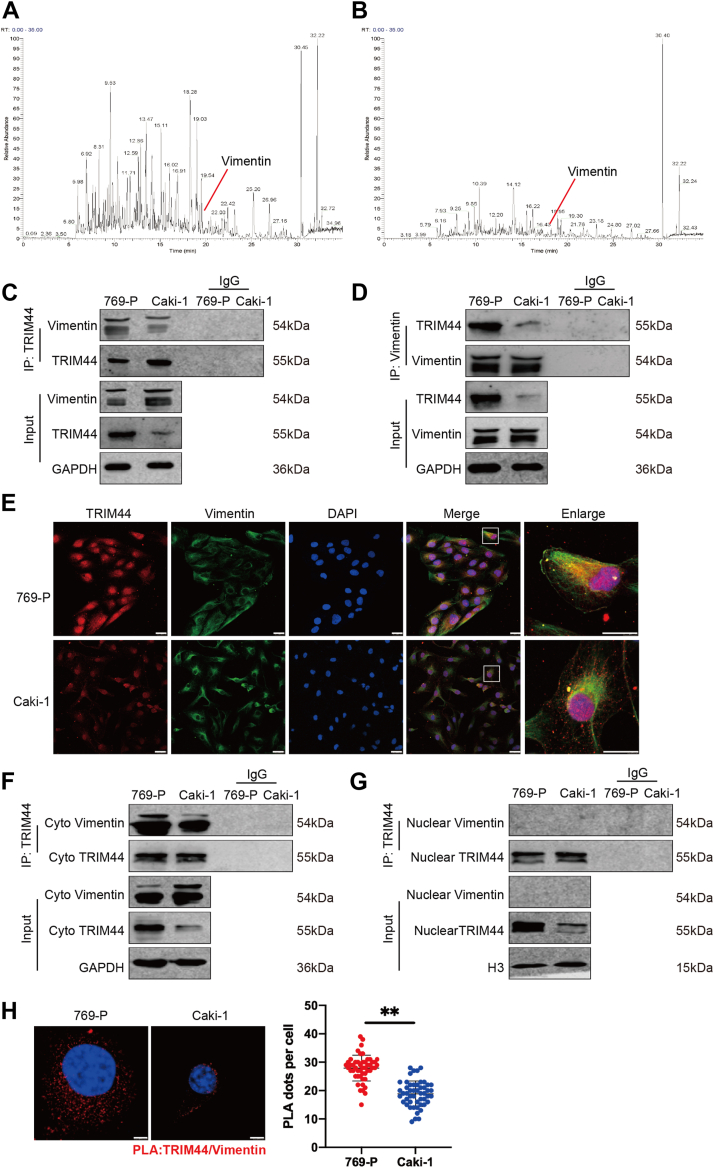
**TRIM44 is bound to vimentin in the cytoplasm in ccRCC cells.***A* and *B*, identification of the interacting protein of TRIM44 by a combination of Co-IP and MS in 769-P and Caki-1 cell lines. *C* and *D*, Validation of TRIM44-vimentin interaction by Co-IP assays in 769-P and Caki-1 cell lines. *E*, IF analysis demonstrating co-localization of TRIM44 and vimentin in 769-P and Caki-1 cell lines. Scale bar: 25 μm. *F* and *G*, the interaction of TRIM44 and vimentin was tested by Co-IP assay in the cytoplasm and nucleus, respectively. *H*, PLA assay was performed to analyze the association of TRIM44/vimentin (*red*) in 769-P and Caki-1 cell lines; the scatter plot showed the average PLA dots per cell. Scale bar: 5 μm. ∗∗*p* < 0.01.

To further elucidate the underlying mechanism of TRIM44 in ccRCC metastasis, we used immunoprecipitation combined with mass spectrometry (MS) and detected hundreds of proteins (Fig. 3, *A* and *B*). Previously, we found that TRIM44 was significantly correlated with the EMT process, we analyzed EMT-related proteins based on dbEMT2.0 database and compared them to our IP-MS result, which suggested that vimentin might have its function in this process. However, vimentin indeed plays an important role as an EMT marker (22, 23) in breast cancer (24), non-small cell lung cancer (25), and other cancers (26, 27).

We firstly verified the interaction between TRIM44 and vimentin by immunoprecipitation and immunofluorescence assays, which showed that TRIM44 interacted with vimentin in 769-P and Caki-1 cell lines (Fig. 3, *C*–*E*), and in OSRC-2 and ACHN cell lines (Fig. S5, *A*–*C*). In addition, TRIM44 is combined with vimentin mainly in the cytoplasm (Fig. 3*E*, S5*C*). We further investigated this interaction by co-immunoprecipitation of isolated nuclear and plasma proteins. TRIM44 and vimentin showed a stronger combination in the cytoplasm in primary ccRCC cell lines (769-P and OSRC-2) than in metastatic ccRCC cells (Caki-1 and ACHN) (Figs. 3F, S5*D*) but showed little combination in the nucleus (Figs. 3*G*, S5*E*). PLAs are used to detect the existence of a direct binding relationship between two proteins (28). TRIM44 interacted directly with vimentin in the cytoplasm (Fig. 3*H*, S5*F*). Collectively, these results suggest that TRIM44 could directly bind to vimentin in the cytoplasm of ccRCC cells.

### TRIM44 negatively regulated vimentin by promoting its degradation *via* ubiquitination

**Figure 4:**
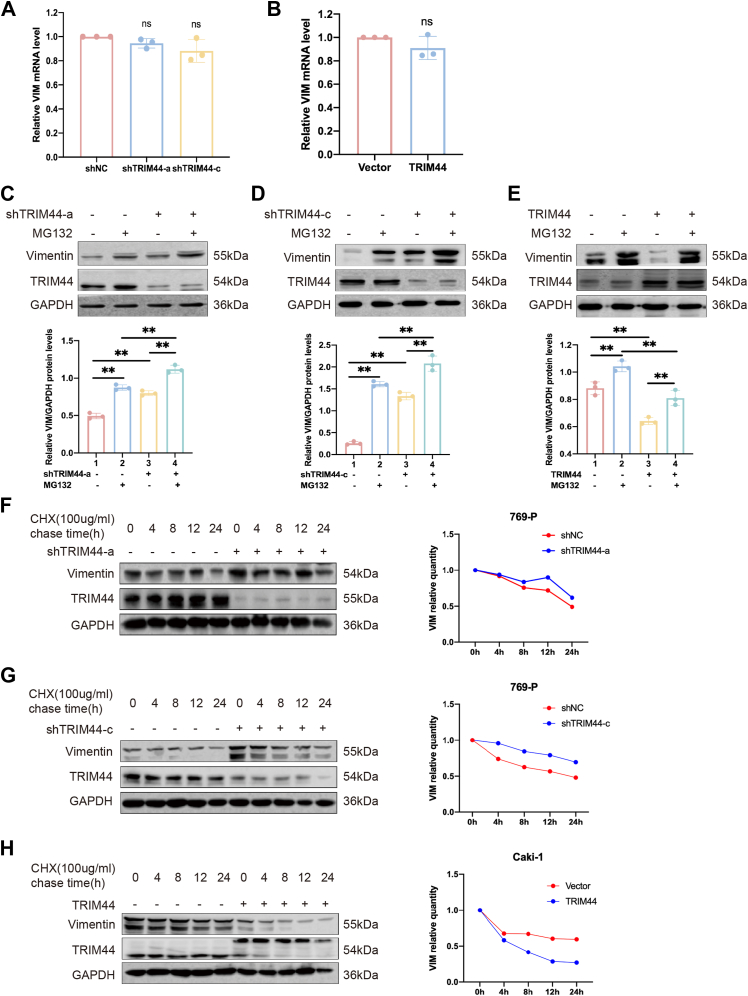
**TRIM44 negatively regulated vimentin by promoting its degradation *via* ubiquitination.***A* and *B*, the mRNA level of vimentin was detected by RT-PCR after TRIM44 interference in 769-P and Caki-1 cells. *C–E*, the expression of vimentin protein was examined by Western blot. *C* and *D*, 769-P cells were transfected with shNC or shTRIM44 plasmid, and 10 μM MG132 for 8 h. *E*, Caki-1 cells were transfected with Vector or TRIM44 plasmid and added 10 μM MG132 for 8 h. *F–H*, the expression level of vimentin was detected by Western blot assay. *F* and *G*, 769-P cells were transfected with shNC or shTRIM44 plasmid and treated with 100 μg/ml CHX. *H,* Caki-1 cells were transfected with the Vector or TRIM44 plasmid and treated with 100 μg/ml CHX. And line charts of the degradation rate of vimentin in 769-P and Caki-1 cell lines. ∗*p* < 0.05; ∗∗*p* < 0.01 and NS, no significance.

We determined the effect of TRIM44 on vimentin expression in ccRCC cells. Vimentin expression was increased after knockdown of TRIM44 in 769-P cells and decreased after TRIM44 overexpression in Caki-1 cells (Fig. 2*E*). Notably, quantitative RT-PCR analysis revealed no corresponding changes in vimentin mRNA levels (Fig. 4, *A* and *B*), suggesting that TRIM44 regulated vimentin protein expression at the post-translational level. We further investigated whether TRIM44 could affect the stability of vimentin using cycloheximide (CHX, a protein synthesis inhibitor) and MG132 (a proteasome inhibitor). Western blot results showed that vimentin expression was increased compared with control, after adding MG132 alone or in combination with TRIM44 knockdown (Fig. 4, *C* and *D*), while vimentin expression decreased after overexpressing TRIM44 together with the addition of MG132 (Fig. 4*E*). CHX could be used to detect the stability of proteins. We therefore added CHX to ccRCC cells at different times, and Western blot results indicated that downregulating TRIM44 enhanced vimentin stability (Fig. 4, *F* and *G*), while overexpressing TRIM44 had the opposite effect (Fig. 4*H*). Considering MG132 may affect autophagy, we co-treated cells with MG132 and the autophagy inhibitor 3-MA. As presented in Figure S6, treatment with 3-MA showed no significant effect on vimentin protein levels regardless of TRIM44 status, in contrast to the marked stabilization observed with MG132 treatment. In summary, TRIM44 regulated vimentin *via* proteasome-mediated degradation.

### TRIM44 targeted vimentin for K48-linked polyubiquitination

**Figure 5:**
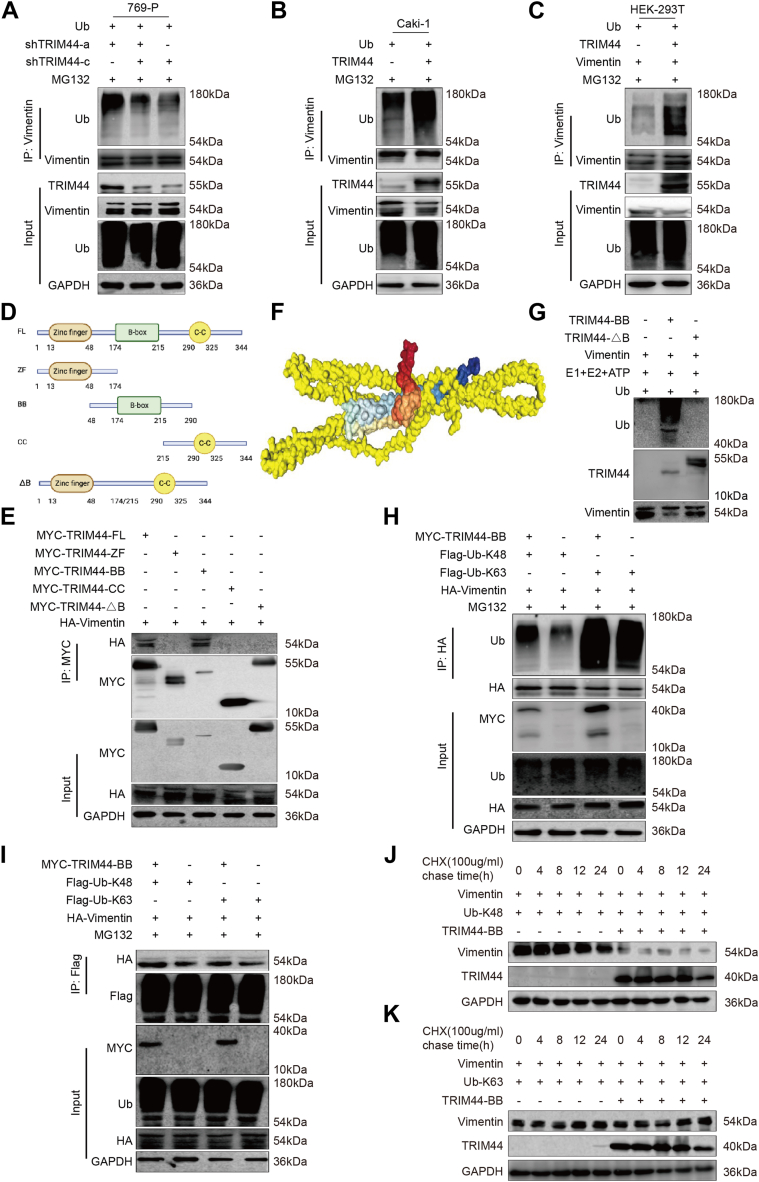
**TRIM44 targeted vimentin for K48-linked polyubiquitination.***A* and *B*, transfecting shNC or shTRIM44 plasmid to 769-P cells, vector or TRIM44 plasmid to Caki-1 cells, and Ub plasmid and MG132 (10 μM for 8 h) to all above cells. The lysates of cells were immunoprecipitated with vimentin antibody and performing Western blot assay with Ub antibody. *C*, HEK-293T cells were co-transfected with vector/TRIM44, vimentin and Ub plasmids, and MG132 (10 μM for 8 h). The lysates of cells were immunoprecipitated with vimentin antibody and performing Western blot assay with Ub antibody. *D*, the schematic diagram of TRIM44 mutant protein. Full-length (FL) TRIM44 (amino acids 1–344); ZF domain, a construct amino acids 13 to 48; BB domain, a construct amino acids 174 to 215; CC domain, a construct amino acids 290 to 325; TRIM44 with a deleted BB domain (△B), a construct without the BB domain (del 174–215). *E*, HEK-293T cells were co-transfected with HA-vimentin and MYC-TRIM44 or TRIM44 deletion mutants. The lysates of cells were immunoprecipitated with MYC-tag antibody and performing Western blot assay with HA-tag antibody. *F*, molecular docking (http://hdock.phys.hust.edu.cn) to predict the binding site between TRIM44 (in *rainbow* color) and vimentin (in *yellow* color). *G*, the *in vitro* ubiquitylation assay was performed by using purified TRIM44-BB, TRIM44-ΔB and vimentin protein in the presence of E1, E2, ubiquitin and ATP. Western blotting was applied to examine the levels of ubiquitin. *H* and *I*, HEK-293T cells were co-transfected with MYC-TRIM44-BB, HA-vimentin, Flag-Ub-K48/K63 and MG132 (10 μM for 8 h). *H*, the lysates of cells were immunoprecipitated with HA-tag antibody and performing Western blot assay with Ub antibody. *I*, the lysates of cells were immunoprecipitated with Flag-tag antibody and performing Western blot assay with HA antibody. *J* and *K*, HEK-293Tcells were transfected with Vector or mutated plasmid with MYC-TRIM44-BB, HA-vimentin and Ub-K48 plasmid (*J*) or Ub-K63 plasmid (*K*), and treated with 100 μg/ml CHX.

Protein degradation occurs *via* the ubiquitin–proteasome system and/or the autophagy–lysosome system (29). The ubiquitin–proteasome system comprises ubiquitin, ubiquitin-activating enzyme (E1), ubiquitin-conjugating enzyme (E2), ubiquitin ligase (E3), and 26S proteasomes (30). The role of TRIM44 in the ubiquitination degradation of vimentin is unknown. We conducted an immunoprecipitation assay to determine if TRIM44 could polyubiquitinate vimentin. Knockdown of TRIM44 decreased the polyubiquitination of vimentin in 769-P cells, while its overexpression increased vimentin polyubiquitination in Caki-1 cells (Fig. 5, *A* and *B*) and HEK-293T cells (Fig. 5*C*, S7*A*), suggesting that TRIM44 can affect polyubiquitination of vimentin protein.

TRIM44 has several known functional domains. We accordingly generated the following mutant TRIM44 protein constructs to identify the domains responsible for binding with vimentin: (i) full-length TRIM44 (FL, 1–344), (ii) TRIM44 with only a zinc finger domain (ZF, 13–48), (iii) TRIM44 with only a BB domain (174–215), (iv) TRIM44 with only a coiled-coil domain (CC, 290–325), and TRIM44 with a deleted BB domain, a construct without the BB domain (△BB, del 174–215) (Fig. 5*D*), and we transfected them into HEK-293T cells. Surprisingly, only the TRIM44 BB domain could bind to vimentin (Fig. 5*E*). To structurally characterize the TRIM44-vimentin interaction, we performed protein-protein docking using the HDOCK server (http://hdock.phys.hust.edu.cn). The HDOCK analysis revealed a preferential binding interface between vimentin (shown in yellow) and TRIM44 (displayed in rainbow colors). Notably, the interaction mainly occurred in the BB domain of TRIM44 and was not related to the ZF and CC domains of TRIM44 (Fig. 5*F*). We also performed the *in vitro* ubiquitination assay by using recombinant TRIM44-BB protein and recombinant TRIM44-ΔB protein to determine if the BB domain of TRIM44 had E3 ligase activity. The results demonstrated its biochemical function as an E3 ligase (Fig. 5*G*).

Ubiquitination modification generally occurs in the protein substrate’s lysine residues, with polyubiquitination at different lysine sites having different functions. For example, lysine-48 (K48)-linked polyubiquitination is mainly related to the degradation of target proteins, while lysine-63 (K63)-linked polyubiquitination could regulate the activation of target proteins (31, 32). To confirm if TRIM44 polyubiquitinated lysine-48 in vimentin, we co-transfected the mutated plasmid with MYC-TRIM44-BB and HA-vimentin into HEK-293T cells. The BB domain of TRIM44-mediated ubiquitination of vimentin relied on K48-linked polyubiquitination (Fig. 5, *H* and *I*). Although high ubiquitination signals on vimentin were observed on K63, we co-transfected HEK-293T cells with the mutated plasmid with MYC-TRIM44-BB, HA-vimentin, and Ub-K48 or Ub-K63, together with CHX, at different times. As shown in Figure 5, *J* and *K*, the degradation of vimentin was unaffected by Ub-K63 but degraded with Ub-K48. Taken together, these findings indicate that the BB domain of TRIM44 is essential for K48-linked polyubiquitination of vimentin.

### TRIM44 correlated with vimentin and affected the progression of ccRCC *in vivo*

**Figure 6:**
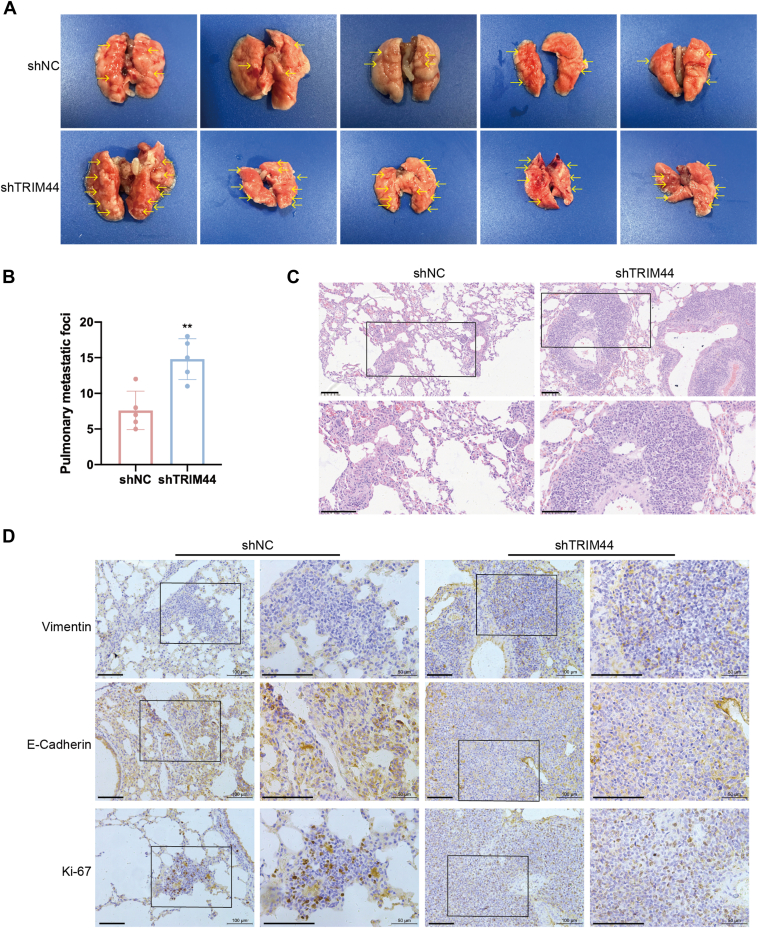
**TRIM44 correlated with vimentin and affected the progression of ccRCC *in vivo*.***A*, lung images of nude mice through tail vein injection of shNC or shTRIM44 derived 769-P cells, n = 5 of each group. And lung images were performed to detect metastatic foci. *B*, statistical data graph of lung metastasis quantification. *C*, representative H&E staining of lung sections. Scale bar: 100 μm. *D*, The expression of vimentin, E-Cadherin, and Ki-67 was edtected by IHC in ccRCC mouse model. Scale bar: 100 μm. ∗∗*p* < 0.01.

To further verify the function of TRIM44 on ccRCC *in vivo*, we constructed 769-P cells stably expressing low levels of TRIM44. We then injected these cells into the tail veins of nude mice to establish a ccRCC metastasis mouse model. Consistent with the cytological results, mice in the shTRIM44 group showed more pulmonary metastatic foci than mice in the shNC group (Fig. 6, *A* and *B*). Hematoxylin and eosin staining confirmed the increase in metastatic sites and greater tumor fusion area in the shTRIM44 group (Fig. 6*C*). Vimentin signals were localized in the cytoplasm, while E-cadherin was located in the cytomembrane and/or cytoplasm, and Ki-67 was located in the nuclei (Fig. 6*D*). Mice in the shTRIM44 group showed increased expression of Ki67 and vimentin and decreased E-cadherin compared with the control group (Fig. 6*C*). We also examined the factors correlated with expression levels in ccRCC clinical samples. We retrospectively examined the expression levels of vimentin, E-cadherin, and Ki-67 proteins in 144 ccRCC tissues by IHC. TRIM44 expression was negatively correlated with vimentin and Ki-67 expression and positively correlated with E-cadherin in the ccRCC group (Fig. 1*D*). We confirmed the above conclusions in 12 pairs of ccRCC and matched peritumoral tissues by Western blot (Fig. 1*F*). Notably, patients with high TRIM44 combined with low vimentin expression levels had the worst prognosis (Fig. 1*H*). In summary, low expression of TRIM44 promoted ccRCC progression *in vivo*.

## Discussion

TRIM44, as a member of the TRIM protein family, has the characteristic properties of its family, but it is also involved in regulating the pathogenesis of various diseases, including tumors, viral infections, and neurodegenerative diseases. For example, TRIM44 aided the progression of breast cancer by promoting cell proliferation, migration, and activation of nuclear factor-κB signaling (33). Knockdown of TRIM44 inhibited the proliferation and invasion of prostate cancer *via* inactivation of the phosphoinositide 3-kinase/Akt pathway (34). Notably, however, the role of TRIM44 in the progression of ccRCC is still unclear. Given the high metastatic propensity of ccRCC, we performed comparative proteomic profiling of primary and metastatic cell lines, which identified TRIM44 as differentially expressed, along with several other proteins known to be involved in renal carcinogenesis. Such as FABP5-mediated ccRCC proliferation through fatty acid metabolism-dependent PI3K/AKT activation (35), MT2A's role in tumor heterogeneity and therapeutic resistance (36), and RIN1's enhancement of ccRCC invasiveness *via* Rab25-dependent EGFR signaling (37). In the present study, we demonstrated that TRIM44 expression was down-regulated in ccRCC, and its level was related to a poor prognosis in patients with ccRCC. Clinically, low TRIM44 expression was associated with larger tumor size, high Fuhrman grade, worse histological grade, advanced TNM, lymph node metastasis, and distant metastasis. Functional assays revealed that TRIM44 overexpression suppressed the invasion, migration, and proliferation of ccRCC cells. Additionally, TRIM44 expression was negatively correlated with EMT, which was characterized by increased expression of vimentin and Snai1 and decreased expression of E-cadherin in ccRCC tissues. Taken together, these results suggest that TRIM44 might play an important role in ccRCC progression by functioning as a tumor suppressor and as a promising biomarker for ccRCC progression.

However, Yamada *et al.* (38) found that TRIM44 could promote the progression of renal cell carcinoma by regulating FRK. The discrepancy between our tumor-suppressive findings and Yamada *et al.*'s reported oncogenic role of TRIM44 in ccRCC stems from two fundamental distinctions. First, our study uniquely demonstrates TRIM44's stage-dependent regulation, paralleling established bifunctional proteins like AP-2 in breast cancer (early proliferation enhanced/advanced stage reduced) (39), TGF-β (early suppressor/late promoter) (40) and TWIST1 (senescence/EMT switch) (41). Although TRIM44 has been reported to exert oncogenic roles in various cancers, such as esophageal carcinoma (17), gastric cancer (14), and multiple myeloma (42), Wu *et al.*’s study also indicate its tumor-suppressive function in squamous cell carcinoma (43). Beyond TRIM44, other TRIM family proteins similarly exhibit such dual roles. For instance, TRIM28 acts as an oncogene in non-small cell lung cancer (NSCLC) (44) but functions as a tumor suppressor in gastric cancer (45). Moreover, our multi-omics integration (proteomic screening coupled with database analysis), functional validation using diverse cell models, and large-scale clinical studies collectively demonstrate TRIM44's tumor-suppressive function in metastatic ccRCC progression.

Different members of the TRIM family have been reported to mediate or promote the occurrence or progression of diseases *via* distinct mechanisms. For example, Yuan *et al.* revealed that TRIM11 promoted viral uncoating to confine human immunodeficiency virus-1 reverse transcription (46). In neoplastic diseases, TRIM15 regulated lipid metabolism *via* the APOA1-LDLR axis to promote pancreatic cancer metastasis (47). TRIM16-induced degradation of vimentin was abrogated by VAL, which directly interacted with vimentin to promote lung adenocarcinoma invasion and metastasis (48). In ovarian cancer, TRIM56 acted as an ubiquitin ligase to target vimentin and inhibit tumor progression (49). Nevertheless, the function of TRIM44 in ccRCC remains unclear. We aimed to clarify the possible mechanism and characteristics of TRIM44 *in vitro*. We performed immunoprecipitation-MS to identify the proteins interacting with TRIM44, among which vimentin was recognized as a marker of EMT. We then changed TRIM44 expression in ccRCC cells and added CHX or MG132, respectively, and examined the effects on vimentin expression. The results showed that TRIM44 regulated and degraded vimentin to prevent ccRCC progression in the cytoplasm. In this context, TRIM44 works as an E3 ligase.

TRIM44 belongs to the unclassified (UC) subgroup of the TRIM family and lacks a RING finger domain but includes a ZF ubiquitin-binding domain, which is involved in the deubiquitination process (50, 51). Yang *et al.* revealed that TRIM44 could bind to VISA and stabilize it by preventing its ubiquitination (52). This suggested that TRIM44 did not have E3 ubiquitin ligase properties, in contrast to the current results. Furthermore, TRIM44 has a BB domain and a CC domain. Several studies indicated that the BB domain could also exert E3 ligase activity (53, 54). For example, TRIM16 acts as an ubiquitin ligase due to its BB domain instead of a classic RING domain (55). Wang *et al.* pointed out that TRIM16 promoted phospho-TAK1 ubiquitination degradation and inhibited the activation of the JNK-p38 pathway to suppress nonalcoholic steatohepatitis progression (56). TRIM29 has a similar structure to TRIM44 without a classic RING domain (50); however, TRIM29 could degrade insulin-like growth factor 2 mRNA-binding protein 1 by inducing its ubiquitination *via* K48-mediated linkage in gastric cancer (57). In theory, TRIM44 can mediate ubiquitination degradation of a protein *via* its BB domain. It is also reported that TRIM proteins may form dimer or oligomer structures; thereby, TRIM44 may interact with other TRIM family proteins to form homo- or heterodimers (58). Interestingly, we reviewed IP-MS results, and there exist other TRIM family members that TRIM44 may interact with. Since our *in vitro* ubiquitination assay result indicated that the TRIM44-BB protein exhibited E3 ligase activity, further suggesting that TRIM44 could work as an E3 ubiquitin ligase. These results thus suggest that TRIM44 may act as an E3 ubiquitin ligase or as a deubiquitin enzyme, depending on the specific domain that binds to the substrate protein.

**Figure 7:**
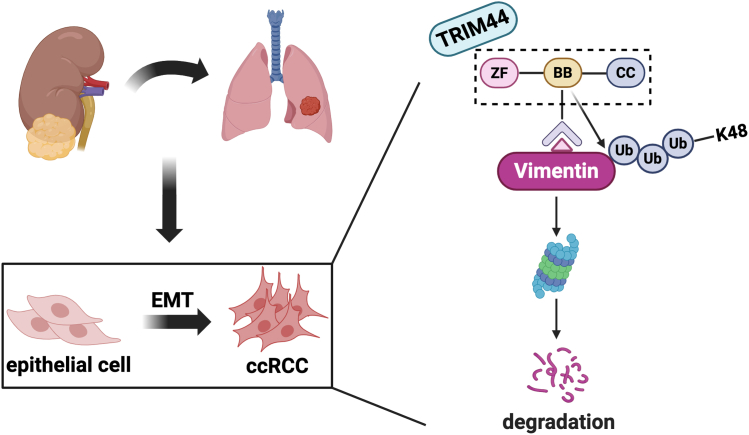
Schematic model illustrating the mechanisms of TRIM44-mediated vimentin ubiquitination regulated EMT and tumor metastasis in ccRCC.

Vimentin is overexpressed in many epithelial cancers and is associated with tumor growth and invasion, and with a poor prognosis (23, 59). Mechanistically, vimentin is a marker of EMT, which is related to several tumorigenic events (60). Notably, vimentin could be regulated by post-translational modifications such as ubiquitination, phosphorylation, and acetylation (49, 61, 62), with its ubiquitination playing an essential role in tumor development. UCHL3 reportedly promoted the migration of hepatocellular carcinoma cells by de-ubiquitinating and stabilizing vimentin (63), whereas vimentin could be regulated by FRMD3 through ubiquitination-mediated degradation in breast cancer, to inhibit tumor growth and metastasis (64). Protein ubiquitination is critical for many cellular processes, such as protein degradation, cell cycle, transcriptional regulation, DNA repair, and signal transduction (5). Ubiquitin is composed of 76 amino acids, which are attached to the target protein through E1 ubiquitin-activating enzyme, E2 ubiquitin-conjugating enzyme, and E3 ubiquitin-ligase (65). In this process, ubiquitin molecules could be added to its lysine residues (including K6, K11, K27, K29, K33, K48, or K63) or the α-amino terminus of the first ubiquitin (32, 65, 66). However, further studies are needed to elucidate the ubiquitination modification of vimentin in ccRCC and the precise mechanism. In the current study, we demonstrated that TRIM44 modulates vimentin stability through its B-box domain and mediates K48-linked polyubiquitination of vimentin (Fig. 7).

This study demonstrated that TRIM44 can bind directly to vimentin and that the BB domain of TRIM44 disrupts the stability of vimentin protein *via* regulating K48-linked ubiquitination to prevent RCC progression. Thus, targeting the TRIM44/vimentin axis might provide a novel strategy for treating ccRCC.

## Experimental procedures

### Patient samples and ethics statement

A total of 144 matched ccRCC and adjacent non-tumorous samples were collected from patients at the Fourth Hospital of Hebei Medical University from 2015 to 2018. All diagnoses were confirmed by histopathological examination. Half of the obtained tissues were snap-frozen and stored in liquid nitrogen and the other half were fixed in 4% formaldehyde. Patient information was obtained from the hospital’s medical record system, and each patient was followed up every 6 months. All patients provided informed consent. The study was approved by the Ethical Review Boards of Hebei Medical University Fourth Affiliated Hospital (2023KS035) and adhered to the principles of the Declaration of Helsinki. All samples were used for experimental research.

### Cell lines and cell culture

The human RCC cell lines (786-O, 769-P, OSRC-2, ACHN, Caki-1, SN12-PM6) were donated by Professor Wang from the Second Hospital of Hebei Medical University. The HEK-293T cell was kept in the laboratory and purchased from the American Type Culture Collection (ATCC). 786-O, 769-P, OSRC-2, and ACHN cells were maintained in RPMI-1640 (Gibco), Caki-1 and SN12-PM6 cells in McCoy's 5A (Gibco) supplemented with 10% fetal bovine serum (FBS, Gibco) and 1% penicillin (Invitrogen), respectively. HEK-293 T cells were cultured in Dulbecco's modified Eagle's medium (DMEM, Gibco) with 10% FBS. Culture conditions were carried out at 37 °C in an incubator (Thermo Fisher Scientific) with 5% CO2 and 95% air.

### Proteomics analysis

Proteins extracted from ccRCC cell lines (786-O, 769-P, OSRC-2, ACHN, Caki-1, SN12-PM6) were enzymatically digested and analyzed by LC-MS/MS. The MS raw data were processed using the MASCOT search engine (v2.2, Matrix Science) integrated into Proteome Discoverer 1.4 for protein identification and quantification, with statistical significance thresholds set at fold-change >1.2 or < 0.8, and *p* < 0.05. All experimental procedures were performed by Shanghai Applied Protein Technology Co., Ltd

### Immunohistochemistry (IHC) staining

ccRCC sections were sequentially fixed in 4% formaldehyde, deparaffinized in xylene, and rehydrated through graded ethanol. Then, using the pressure cooker to recover the antigen and block the endogenous peroxidase through 3% H_2_O_2_ for 30 min at room temperature. Next, slides were blocked with 10% goat serum and incubated with primary antibodies (listed in Table S2) overnight at 4 °C in turn. On the second day, slides were incubated with polymer helper and polyperoxidase anti-mouse/rabbit IgG at 37 °C and finally stained with diaminobenzidine (DAB). Finally, an Olympus microscope (OLYMPUS, BX71, Tokyo, Japan) was used to capture images. The average integrated optical density value was quantified to indicate the expression of protein by Image Pro Plus (Media Cybernetics). The colorectal cancer section was selected as the positive control, while the negative control was set up using PBS rather than the primary antibody.

### Plasmid and transfection

Flag-TRIM44, Myc-TRIM44, Myc-TRIM44-ZF, Myc-TRIM44-BB, Myc-TRIM44-CC, Myc-TRIM44-ΔB, HA-vimentin, Flag-Ub plasmids, Flag-Ub-K48, and Flag-Ub-K63 were purchased from YouBio by cloning the corresponding human full-length DNA sequence into the MYC or HA or 3xFlag-pcDNA 3.1(+), and its mutant expression plasmid was generated by in-fusion cloning kits. Specifically, Flag-Ub-K48 and Flag-Ub-K63 mutants were generated by mutating all ubiquitin lysine residues to arginine (R) except for K48 or K63, respectively. Three shRNAs of TRIM44 were constructed and selected according to the knockdown efficiency (Fig. S2). All mutation primers were listed in Table S1. Transfection experiments using Lipofectamine 3000 reagents (Invitrogen, Carlsbad, CA, USA, #L3000015) under the manufacturer's protocols.

### Viruses and transduction

Lentivirus of shTRIM44 and a negative control were obtained from Hanbio. The transfection methods were performed according to the manufacturer's protocols. The cell lines were treated with puromycin for 2 weeks to obtain a stably expressed cell line.

### Western blot assay

Total protein from ccRCC and HEK-293T cells or the tissue from patients with ccRCC was extracted with RIPA lysis buffer. An equal amount of protein extracts (30 μg) was separated by 10 or 12% SDS-PAGE and then transferred to PVDF membranes (Millipore). The membranes were blocked with 5% nonfat milk for 1.5 h at 37 °C. Then the membranes were incubated overnight at 4 °C with primary antibodies (listed in Table S2). On the second day, the membranes were incubated with a horseradish peroxidase-conjugated goat anti-rabbit or mouse IgG antibody. After washing, the images were captured using the LI-COR Odyssey Infrared Imaging System. All experiments were repeated at least three times.

### Cell wound scratch assay

A straight line was drawn by a sterile 200 μl pipette tip in the 6-well plates. Then, replace the medium with an FBS-free medium and measure the initial and unhealed width. Each of the wells was randomly selected from three different visual fields three times, and the average value was taken.

### Transwell migration and invasion assay

Cell migration and invasion were measured using a two-chamber transwell system (8 μm pore size). ccRCC cells (5 × 10^4^) were seeded into upper chambers using an FBS-free medium. The lower chamber was filled with medium supplemented with 10% FBS. After co-culturing at 37 °C for 24 h, the lower surface of the upper chambers was fixed with 4% paraformaldehyde for 30 min and stained with crystal violet for 30 min at room temperature. As for the invasion assay, the procedure was performed as described above with the upper chamber membrane precoated with Matrigel (Corning).

### Colony formation assay

A total of 4000 transfected cells were seeded into the 6-well plates and incubated at 37 °C with 5% CO_2_ for 14 days. Using 4% paraformaldehyde to fix cells in the 6-well plates for 20 min and staining with crystal violet for 15 min. The Image Pro Plus (Media Cybernetics, Silver Spring, MD) was used to measure the colony.

### Immunofluorescence and EdU proliferation staining

The ccRCC cells were inoculated in 6-well plates, fixed with 4% paraformaldehyde for 30 min at room temperature, and lysed with 0.3% Triton X-100 for 15 min at room temperature. Then, cells were blocked with 10% goat serum at 37 °C for 1 h and incubated with primary antibodies (listed in Table S2) at 4 °C overnight. The next day, cells were incubated with FITC-conjugated goat anti-rabbit IgG/TRITC-conjugated goat anti-mouse IgG at 37 °C for 2 h followed by treatment with 4′,6-diamidino-2-phenylindole (DAPI; Southernbiotech). The sections were observed with a Leica Laser Confocal Microscope (Leica). The ccRCC cell proliferation level was evaluated by measuring the incorporation of EdU using the EdU Cell Proliferation Kit (Beyotime Biotechnology) according to the manufacturer's instructions. Briefly, adding EdU (10 μM) to the culture medium for 2 h before collecting cells, then following the Immunofluorescence steps described above, and using a Lecia Laser Confocal Microscope (Leica) to observe the positive signal, which was located in nuclei and red granular, and the positive ratio was quantified by Image Pro Plus (Media Cybernetics).

### Real-time PCR assay

Total RNA of cells was extracted using TRIzol reagent (Invitrogen). RNA concentration was measured by NanoDrop 2000. Reverse transcription was performed by HiScriptⅢ RT SuperMix for qPCR (Vazyme, R323), and polymerase chain reaction (qPCR) was performed with ChamQ Universal SYBR qPCR Master Mix (Vazyme, Q711) following the manufacturer's instructions. The 2^−ΔΔCT^ method was used to normalize the qPCR cDNAs. All experiments were repeated at least in triplicate.

### Immunoprecipitation and MS

Cells were lysed with RIPA lysis buffer for 40 min, and the proteins were quantified using a BCA assay kit. The lysate (800 μl) was incubated with primary antibodies (listed in Table S2) on a mixer for 24 h at 4 °C and then incubated with protein A/G beads (20 μl) on the mixer at 4 °C overnight. The samples were then washed three times with immunoprecipitation washing buffer at 500*g* for 5 min at 4 °C. Bromophenol blue protein indicator buffer was added, and the extracted proteins were boiled at 100 °C for 7 min.

For IP-MS, cell lysates from 769-P and Caki-1 cells were immunoprecipitated with anti-TRIM44 antibody, separated by SDS-PAGE, and stained with Coomassie blue. Target bands were excised for in-gel trypsin digestion and subsequent LC-MS/MS analysis by Shanghai Applied Protein Technology Co., Ltd.

### Proximity ligation assay

A Duolink Proximity Ligation Assay kit (Sigma, DUO92101) was used to detect direct binding relationships between proteins. First, 1 × 10^6^ cells were seeded into 6-well plates. On the second day, the cells were fixed with 4% paraformaldehyde solution, permeabilized with 0.3% Triton X-100, blocked with Duolink Blocking Solution, and probed with antibodies directed against TRIM44 (1:100) and vimentin (1:500) at 4 °C overnight. The following day, the cells were incubated with *in situ* PLA probes anti-mouse plus and anti-rabbit minus (diluted 1:5) at 37 °C for 1 h. The cells were then washed with Buffer A solution, and the Ligation buffer was added and incubated at 37 °C for 30 min, followed by two washes. Amplification buffer was then added and incubated at 37 °C for 100 min, and the cells were washed three times with Wash Buffer B solution and once with 0.01× diluted Wash Buffer B solution. Cells were mounted using Mounting Medium with DAPI, and images were taken under a Leica Laser Confocal Microscope (Leica).

### *In vitro* ubiquitination assay

Recombinant TRIM44-BB protein (GENEWIZ) and TRIM44-ΔB protein (GENEWIZ) used in this study were commercially synthesized and produced in a mammalian expression system (HEK293T cells), while recombinant vimentin protein (Sino Biological, 10,028-H08 B) was produced in the baculovirus system (Sf9 insect cells). Recombinant vimentin protein and recombinant TRIM44 protein were incubated with E1 Enzyme (UBE1), E2 Enzyme (UBE2D3, also known as UBCH5C), Ubiquitin, and ATP-Mg solution of a ubiquitination kit (Yeasen, 20439ES20) for 3 h at 37 °C, and the resulting reaction mixtures were used for Western blot analysis.

### Animal study

All animal experiments were performed with the approval of the Committee on Ethics of Biomedicine, Hebei Medical University (2023KY081). Cells (5 × 10^6^ cells/mouse) in 100 μl of phosphate-buffered saline (769-P NC, 769-P shTRIM44) were injected into the tail vein of 5-week-old male BALB/c nude mice (HFK BIOSCIENCE). The mice were euthanized, and the lungs were dissected to count the number of pulmonary metastatic foci. All mice were randomized to either the 769-P NC or 769-P shTRIM44 group, and the investigators were blinded to the group assignment.

### Haematoxylin-eosin (HE) staining

Tissues were fixed in 4% paraformaldehyde solution and using the gradient ethanolxylene to dehydrate and transparent. Then immersing in wax and embedding, sectioning into 2-μm slides, deparaffinizing after drying, and placing in hematoxylin-iridine stain in red solution, washing with water after dyeing, dehydrating with gradient ethanol, transparent xylene, and mounting with neutral gum. Finally, observing the histopathological changes through the microscope.

### Statistical analysis

The results were expressed as the mean ± standard deviation. The data were analyzed using SPSS 25.0 (SPSS, Inc.) and GraphPad Prism 8 software (GraphPad Software). Variables were compared between the two groups using Student’s and paired samples *t*-tests. One-way analysis of variance (ANOVA) was performed to evaluate the significance of differences among multiple groups by Bonferroni’s correction. Survival analysis was carried out using the Kaplan–Meier method and analyzed by the log-rank test. Independent prognostic factors were analyzed by Cox’s proportional hazards regression model. In this study, *p* < 0.05 was considered statistically significant.

## Data availability

The data used and analyzed in this study are available from the corresponding author upon request. The mass spectrometry proteomics data have been deposited to the ProteomeXchange with identifiers PXD065914 and PXD065922.

## Supporting information

This article contains supporting information.

## Ethical statement

The study was approved by the Ethical Review Boards of Hebei Medical University Fourth Affiliated Hospital (2023KS035). All patients signed informed consent forms. And animal experiments were approved by the Institutional Animal Care and Use Committee of Hebei Medical University (2023KY081).

## Conflict of interest

The authors declare that they do not have any conflicts of interest with the content of this article.
