# Supplementary material for: Loss of TRIM44 promotes renal cell carcinoma progression by regulating K48-linked ubiquitination of vimentin
Source: J Biol Chem. 2025 Sep 16;301(11):110734. doi: 10.1016/j.jbc.2025.110734 (PMC12547515; doi:10.1016/j.jbc.2025.110734)
Supplement: Supporting information [file mmc1.docx]

# Supporting Information

**Supplemental figures and legends**

**Fig. S1**

**Fig. S
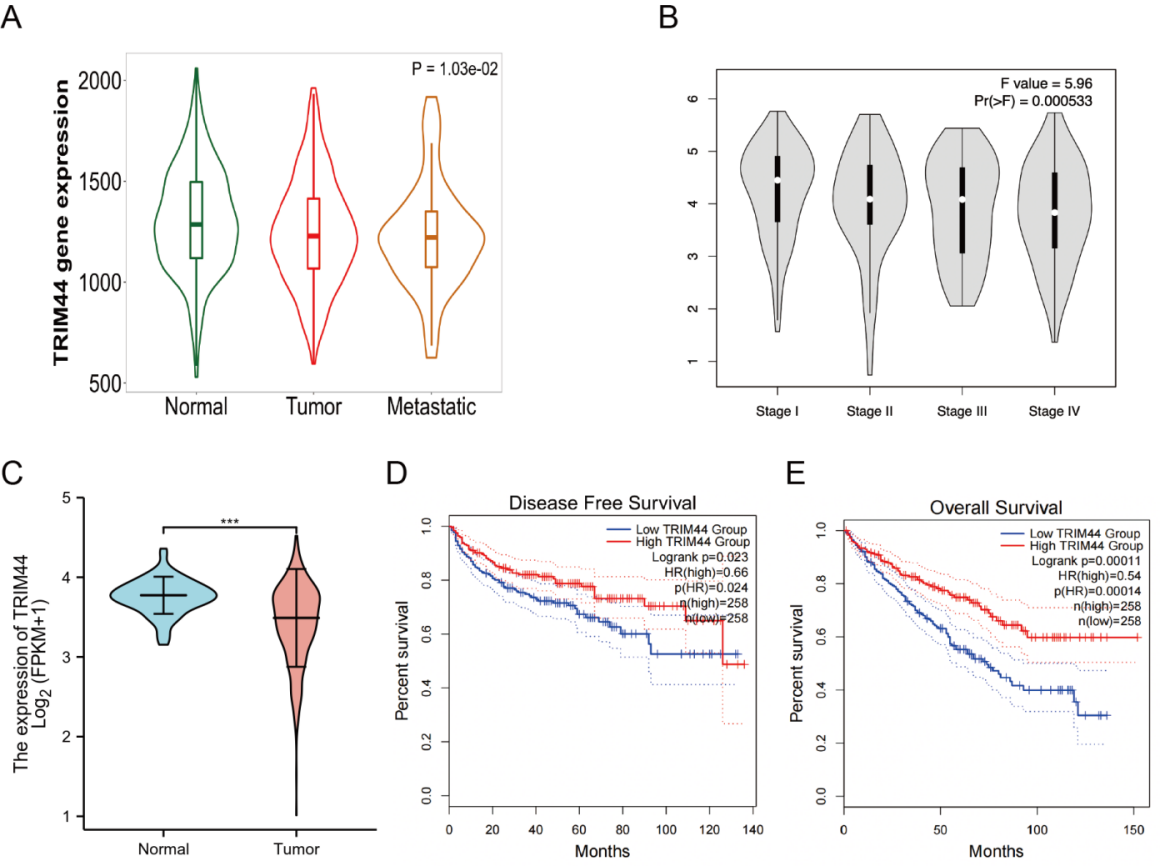
1: Decreased TRIM44 associated with worse prognosis in renal cell carcinoma.**

1. TNMplot database indicated the expression of TRIM44 in normal, tumor and metastatic lesions. (B) TCGA database declared TRIM44 was related to tumor stage. (C) TCGA database displayed the expression of TRIM44 in ccRCC and normal tissue. (D-E). Kaplan–Meier analysis showed around 10-year disease-free survival and overall survival for ccRCC patients. ****P* < 0.001; HR, hazard ratio.

**Fig. S2**

**Fig.
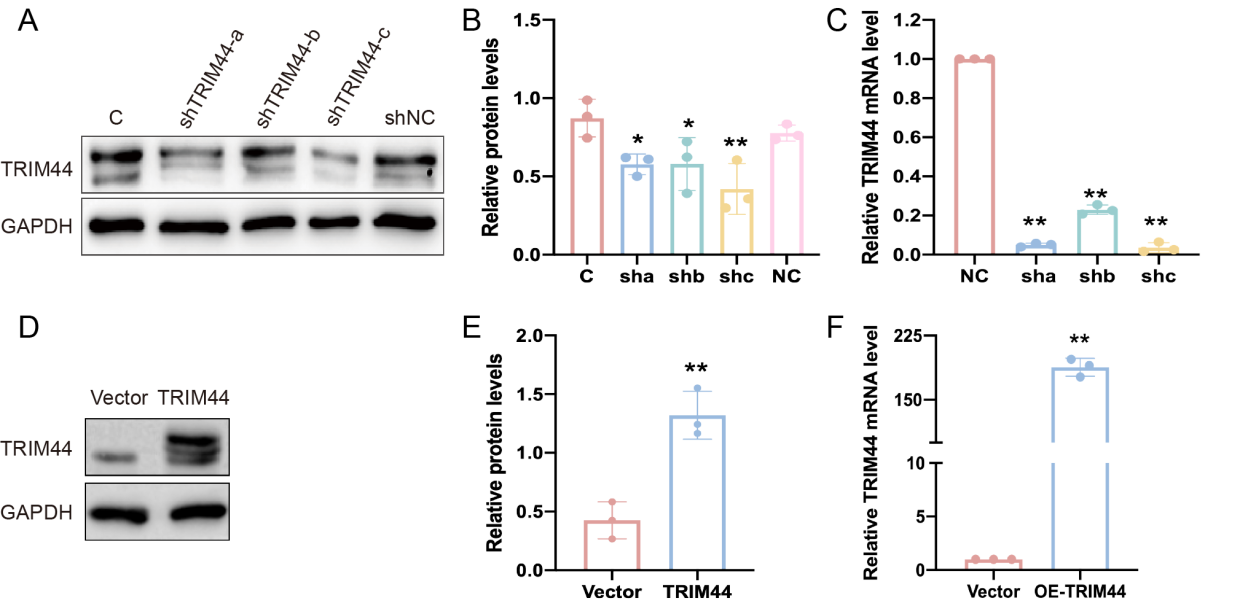
S2: Transfection efficiencies of TRIM44 interference.**

(A-C) The efficiencies of TRIM44 inhibition were examined by western blot assay and RT-PCR. (D-F) The efficiencies of TRIM44 overexpression were examined by western blot assay and RT-PCR. **P* < 0.05 and ***P* < 0.01.

# Fig. S3

# **Fig. S**
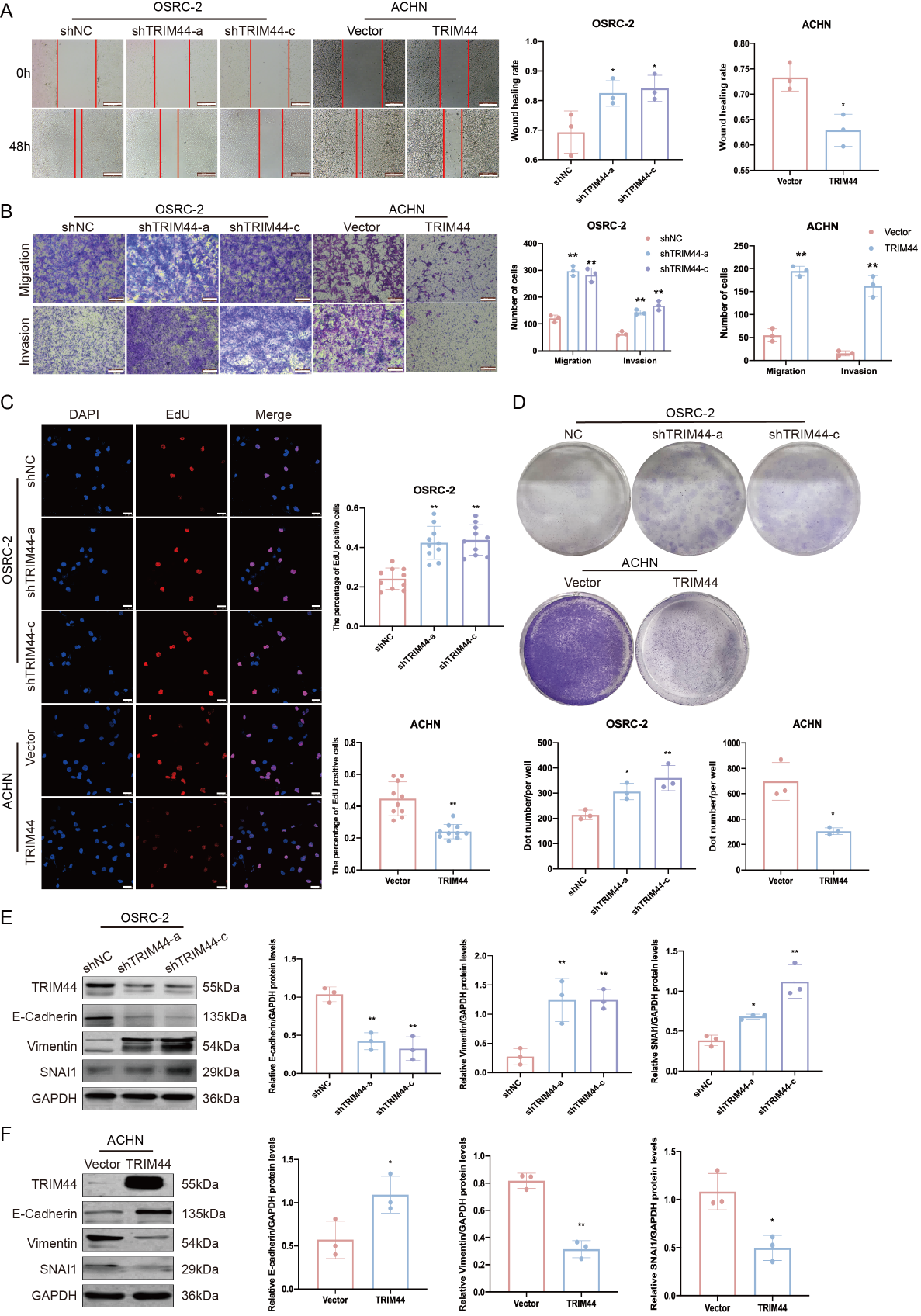
3: **TRIM44 restrained ccRCC progression *in vitro.***

(A-D) Knockdown of TRIM44 enhanced cell migration, invasion, proliferation and colony formation ability in OSRC-2 cell lines, overexpression of TRIM44 reduced cell migration, invasion, proliferation and colony formation ability in ACHN cell lines. (A) Cell wound scratch assay. Scale bar: 500 μm. (B) Transwell migration and invasion assay. Scale bar: 100 μm.(C) EdU proliferation staining, Scale bar: 25 μm. (D) Colony formation assay. (E-F) The expression of E-Cadherin, vimentin, snai1 and TRIM44 was detected by western blot assay. **P* < 0.05 and ***P* < 0.01.

# Fig. S4

# **Fig. S
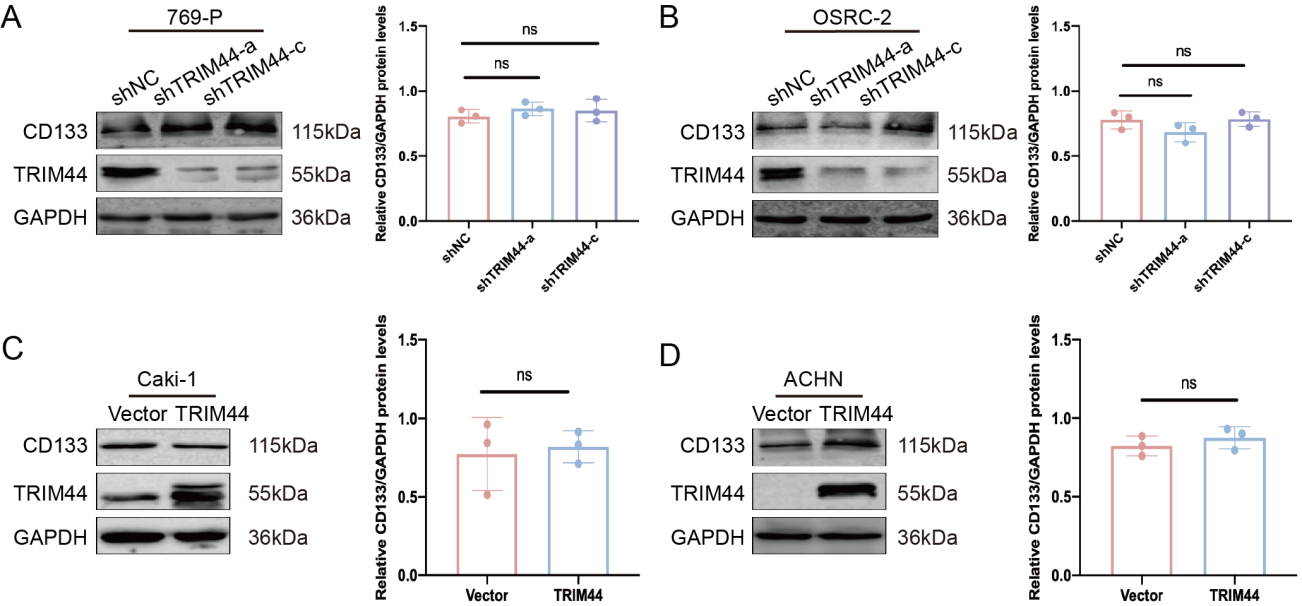
**4**: TRIM44 interference was not associated with stemness in ccRCC.**

1. D) Western blot assay was used to detect the expression of stemness marker (CD133) after TRIM44 interference. (A) In 769-P cell lines, (B) in OSRC-2 cell lines, (C) in Caki-1 cell lines, (D) in ACHN cell lines. NS, no significance.

# **Fig. S5**

#
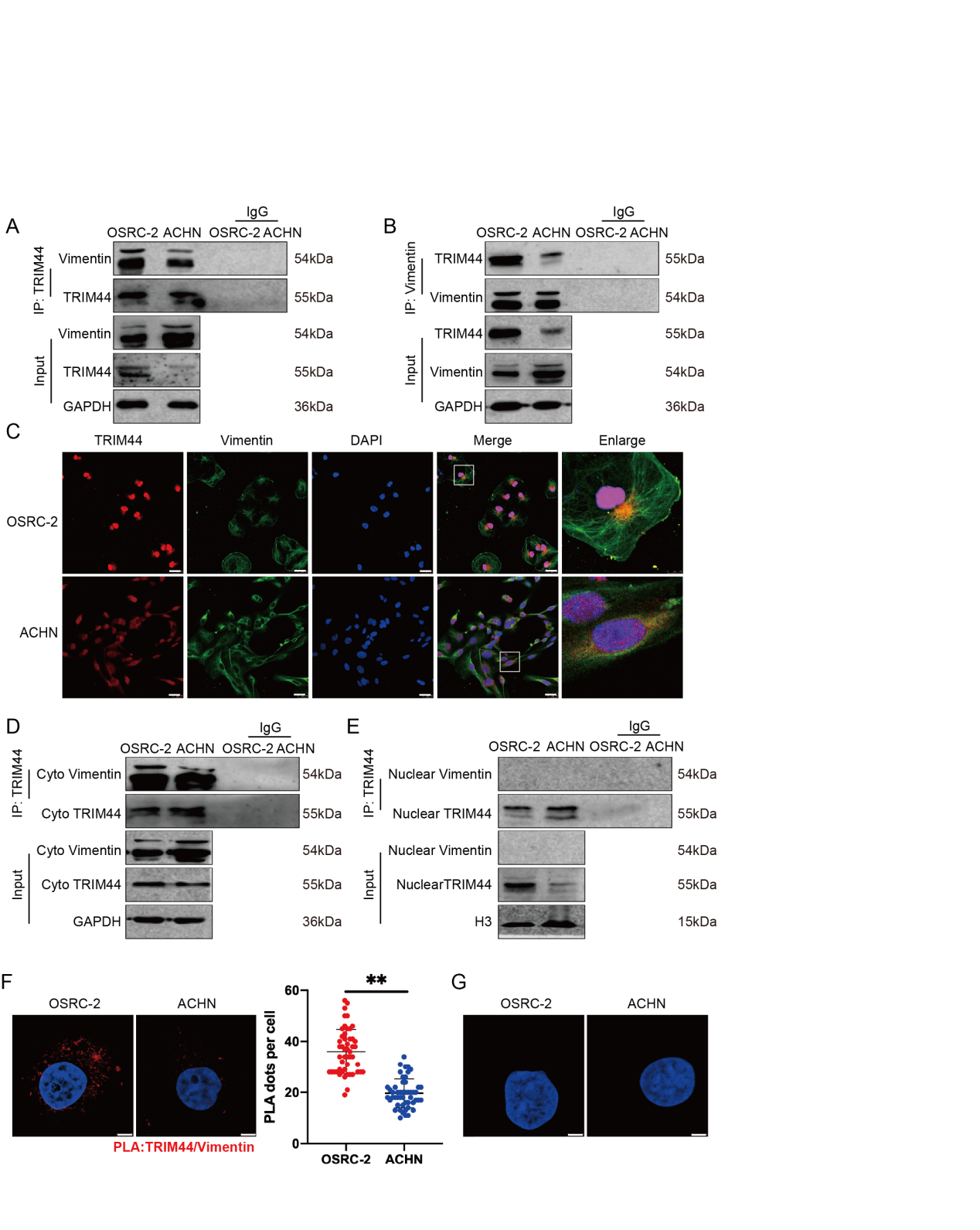
**Fig. S5: TRIM44 bound to vimentin in the cytoplasm in ccRCC cells.**

(A-B) The interaction of TRIM44 and vimentin was tested by Co-IP assay in OSRC-2 and ACHN cell lines, respectively. (C) The co-localized of TRIM44 and vimentin was examined by IF assay in OSRC-2 and ACHN cell lines. Scale bar: 25 μm. (D-E) The interaction of TRIM44 and vimentin was tested by Co-IP assay in the cytoplasm and nuclear. (F) PLA assay was performed to analysis the association of TRIM44/vimentin (red) in OSRC-2 and ACHN cell lines, the scatter plot showed the average PLA dots per cell. (G) The negative control (single-antibody controls with anti-TRIM44 alone) of PLA assay in OSRC-2 and ACHN cells. Scale bar: 5 μm. **P < 0.01.

**Fig. S6**


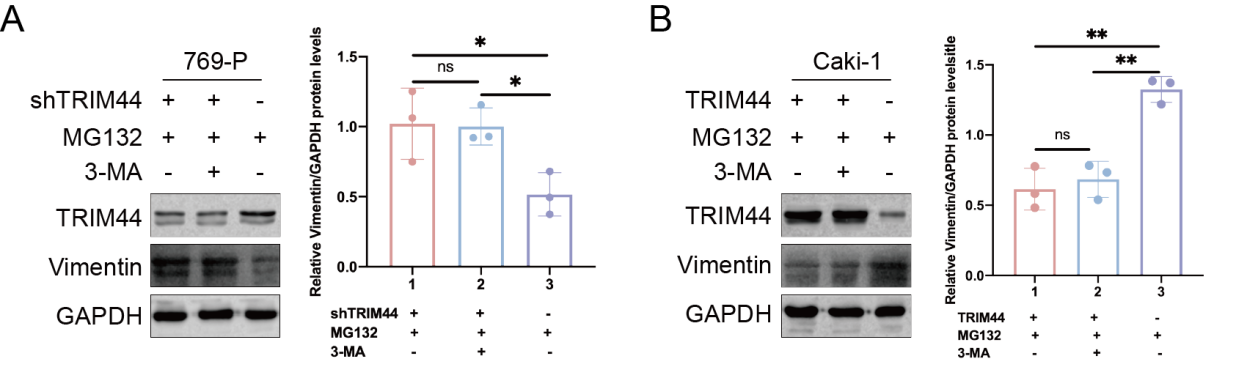
**Fig. S6: TRIM44 regulates vimentin stability through the proteasome pathway rather than autophagy.**

1. B) Western blot analysis of vimentin expression in 769-P (A) and Caki-1 (B) cells treated with the proteasome inhibitor MG132 (10 μM for 8 h) and/or autophagy inhibitor 3-MA (5 mM for 24 hours), with or without TRIM44 modulation.

**Fig. S7**

**
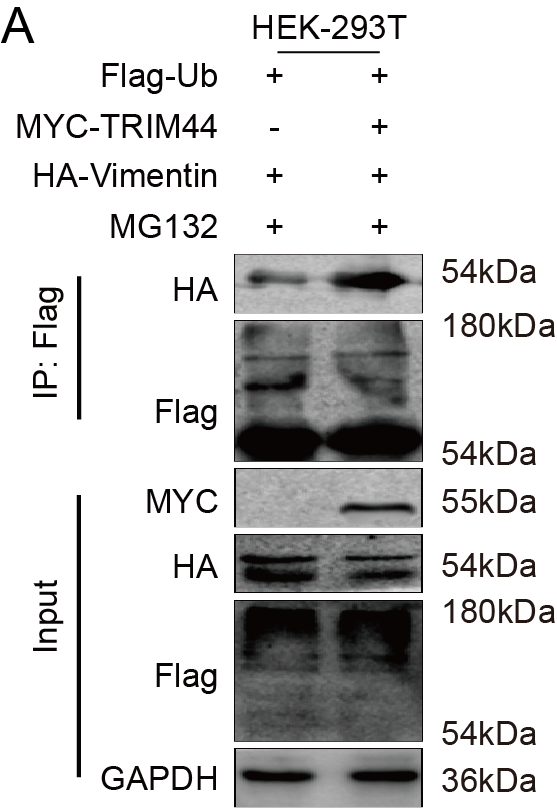
Fig. S7: TRIM44 affects polyubiquitination of vimentin protein.**

1. HEK-293T cells were co-transfected with Flag-Ub, vector/MYC-TRIM44 and HA-vimentin plasmids, and MG132 (10μM for 8 h). The lysates of cells were immunoprecipitated with anti-Flag antibody and performing western blot assay with anti-HA antibody.

# Supplementary Tables

# Supplementary **Table S1: The sequences of primer and shRNA.**

| **Name** | **Sequence** |
| --- | --- |
| TRIM44 forward | 5’-TGAGGCAGAAAGTGAATTTGAC-3’ |
| TRIM44 reverse | 5’-CTTCCTGGCAATAGGTACTCAA-3’ |
| Vimentin forward | 5’-TTGAACGCAAAGTGGAATC-3’ |
| Vimentin reverse | 5’-AGGTCAGGCTTGGAAACA-3’ |
| GAPDH forward | 5’-AGGTCGGTGTAGACGGATTTG-3’ |
| GAPDH reverse | 5’-TGTAGACCATGTAGTTGAGGTCA-3’ |
| shTRIM44-a | 5’-GGCTTGATTTGAGTACCTATT-3’ |
| shTRIM44-b | 5’-GGAACAACTTGATACCTCTAA-3’ |
| shTRIM44-c | 5’-GCTCAGCAACAAACGTACTTC-3’ |

# Supplementary **Table S2: Antibodies used in this study.**

| **Name** | **Manufacturer** | **Cat number** | **Application** |
| --- | --- | --- | --- |
| TRIM44 | Proteintech | 11511-1-AP | 1:50 for IHC; 1:1000 for WB;  1:100 for IF; 2 µg for IP |
| Vimentin | Proteintech | 60330-1-Ig | 1: 5000 for IHC; 1:20000 for WB;  1:200 for IF; 2 µg for IP |
| E-Cadherin | Abcam | ab231303 | 1: 200 for IHC; 1:1000 for WB |
| Ki-67 | Abcam | Ab16667 | 1:200 for IHC |
| Snai1 | Proteintech | 13099-1-AP | 1:500 for WB |
| CD133 | Abcam | Ab284389 | 1:1000 for WB |
| Histone H3 | Proteintech | 17168-1-AP | 1:8000 for WB |
| Ubiquitin | Santa | sc-271289 | 1:500 for WB |
| MYC-Tag | Proteintech | 16286-1-AP | 1:8000 for WB; 2 µg for IP |
| HA-Tag | Proteintech | 66006-2-Ig | 1:20000 for WB; 2 µg for IP |
| Flag-Tag | Affinity | #T0003 | 1:10000 for WB; 2 µg for IP |
| GAPDH | Proteintech | 10494-1-AP | 1:10000 for WB |
| IgG | Santa | sc-2025 | 2 µg for IP |

IHC: Immunohistochemistry; WB: Western blotting; IP: Immunoprecipitation; IF: Immunofluorescence; Proteintech (Hubei, China); Abcam (Cambridge, UK); Affinity (Jiangsu, China); Santa (Texas, USA).
